# Supplementary material for: foxr1 is a novel maternal-effect gene in fish that is required for early embryonic success
Source: PeerJ. 2018 Aug 23;6:e5534. doi: 10.7717/peerj.5534 (PMC6109588; doi:10.7717/peerj.5534)
Supplement: Supplemental Information 1 — Species, database, accession number and amino acid sequence are shown. [file peerj-06-5534-s001.pdf]

Supplemental Data 1. List of f*oxR1* -related protein sequences used in the phylogenetic analysis.

Species common name	Species latin name	Database	Sequence ID	Annotation	
Human	*Homo sapiens*	ENSEMBL v.91	ENSG00000176302	*foxr1*	MGNELFLAFTTSHLPLAEQKLARYKLRIVKPPKLPLEKKPNPDKDGPDYEPNLWMWVNPNIVYPPGKLEVSGRRKREDLTSTLPSSQPPQKEEDASCSEAAGVESLSQSSSKRSPPRKRFAFSPSTWELTEEEEAEDQEDSSSMALPSPHKRAPLQSRRLRQASSQAGRLWSRPPLNYFHLIALALRNSSPCGLNVQQIYSFTRKHFPFFRTAPEGWKNTVRHNLCFRDSFEKVPVSMQGGASTRPRSCLWKLTEEGHRRFAEEARALASTRLESIQQCMSQPDVMPFLFDL
Human	*Homo sapiens*	ENSEMBL v.91	ENSG00000189299	*foxr2*	MDLKLKDCEFWYSLHGQVPGLLDWDMRNELFLPCTTDQCSLAEQILAKYRVGVMKPPEMPQKRRPSPDGDGPPCEPNLWMWVDPNILCPLGSQEAPKPSGKEDLTNISPFPQPPQKDEGSNCSEDKVVESLPSSSSEQSPLQKQGIHSPSDFELTEEEAEEPDDNSLQSPEMKCYQSQKLWQINNQEKSWQRPPLNCSHLIALALRNNPHCGLSVQEIYNFTRQHFPFFWTAPDGWKSTIHYNLCFLDSFEKVPDSLKDEDNARPRSCLWKLTKEGHRRFWEETRVLAFAQRERIQECMSQPELLTSLFDL
Human	*Homo sapiens*	ENSEMBL v.91	ENSG00000139445	*foxN1*	MIESDTSSIMSGIIRNSGQNHHPSPQEYRLLATTSDDDLPGDLQSLSWLTAVDVPRLQQMASGRVDLGGPCVPHPHPGALAGVADLHVGATPSPLLHGPAGMAPRGMPGLGPITGHRDSMSQFPVGGQPSSGLQDPPHLYSPATQPQFPLPPGAQQCPPVGLYGPPFGVRPPYPQPHVAVHSSQELHPKHYPKPIYSYSCLIAMALKNSKTGSLPVSEIYSFMKEHFPYFKTAPDGWKNSVRHNLSLNKCFEKVENKMSGSSRKGCLWALNLARIDKMEEEMHKWKRKDLAAIHRSMANPEELDKLISDRPESCRRPGKPGEPEAPVLTHATTVAVAHGCLAVSQLPPQPLMTLSLQSVPLHHQVQPQAHLAPDSPAPAQTPPLHALPDLSPSPLPHPAMGRAPVDFINISTDMNTEVDALDPSIMDFALQGNLWEEMKDEGFSLDTLGAFADSPLGCDLGASGLTPASGGSDQSFPDLQVTGLYTAYSTPDSVAASGTSSSSQYLGAQGNKPIALL
Human	*Homo sapiens*	ENSEMBL v.91	ENSG00000053254	*foxN3*	MGPVMPPSKKPESSGISVSSGLSQCYGGSGFSKALQEDDDLDFSLPDIRLEEGAMEDEELTNLNWLHESKNLLKSFGESVLRSVSPVQDLDDDTPPSPAHSDMPYDARQNPNCKPPYSFSCLIFMAIEDSPTKRLPVKDIYNWILEHFPYFANAPTGWKNSVRHNLSLNKCFKKVDKERSQSIGKGSLWCIDPEYRQNLIQALKKTPYHPHPHVFNTPPTCPQAYQSTSGPPIWPGSTFFKRNGALLQDPDIDAASAMMLLNTPPEIQAGFPPGVIQNGARVLSRGLFPGVRPLPITPIGVTAAMRNGITSCRMRTESEPSCGSPVVSGDPKEDHNYSSAKSSNARSTSPTSDSISSSSSSADDHYEFATKGSQEGSEGSEGSFRSHESPSDTEEDDRKHSQKEPKDSLGDSGYASQHKKRQHFAKARKVPSDTLPLKKRRTEKPPESDDEEMKEAAGSLLHLAGIRSCLNNITNRTAKGQKEQKETTKN

Mouse	*Mus musculus*	ENSEMBL v.91	ENSMUSG00000074397	*foxr1*	MGNESFLAFTTAHLPLAEQNLARYKLRVVDPPKLPLEKKPNSEDDGPEFEPNLWMWVNPNVVFPPGKLEVPESSKREDVASTLPSPQPPQEEEDANGSEATGVESLPLSSSEQSPSQKHFASSPSTLELTEEEEAEDQDDGSSVALPSPHKRAPLQNRRLRQANNQAGRLWSRPPLNYFHLIALALRNSSPCGLNVQQIYSFTRHHFPFFRTAPEGWKNTVRHNLCFRDSFEKVPVSAQGGANTRPRSCLWKLTEEGHRRFEEEARALASTRLQSIQQCMSQPDVMPFLFDL
Mouse	*Mus musculus*	ENSEMBL v.91	ENSMUSG00000071665	*foxr2*	MDVKVKNRDFWYSLHGQVPGMLDWDMGNEFFLPCTMDQCSFAEQSLAKYKIQLTKPPALPQKKKSNFDDDGPPAEPSLWMWVNPNIVCPINSKEAPNTIHKILPSAPFPQTGESDYLGTQRMVQSLSVLHTEHHQQQKLLIYSTAPDFIEEETKEQECTSSKKYSKKHTVCHGPHREKESWPRPPLNYSHLVALALKSSPSCGLNVQQIYNFTRQHFPYFRTAPEGWKNTIRHNLCSLTCFEKVPVDLEDEPDGKPRSFLWKLTDEGNRFFQEDTRVLAYARRESIKQSMRQPELIDLLFHL
Rat	*Ratus norvegicus*	ENSEMBL v.91	ENSRNOG00000029089	*foxr1*	MGNECFLSFTTSHLSEAEQKLALYRLRIVEPPKLPLEKIPNPDKDGPDIKPNLWMWVNPNIVYPPGKLEVAVKKEDSSSAPPASQSALKEEDSCSEASEIPAQQPPPPRKQKRKQRRNTLPLPLASGRRAPLQNRWRLRQAISPEGRLWSRPPLHYFHLIALALRNSPPCGLSVQQIYNFTREHFPFFRTAPEAWKNTVRHNLCFRDSFEKVPVSKQGEASTRPRSCLWKLTEEGHRRFSEEARTLASTRLQSIQQCMSQPGVKP
Rat	*Ratus norvegicus*	ENSEMBL v.91	ENSRNOG00000030619	*foxr2*	MNVKVKNRDFWYSLHGQVPGMLDWDMGNEFFLPCTMDQCSFAEQSLAKYKIQLTKPPTLPQEKKSSFDNDGPPTEPSLWMWVNPNIVCPVNSKEPPNPIHKVLPSAAFPQTGESECFGTQRVAQSLSVIHTEYHQQQKLPIYSTVPHFMEEETKEQECASSKKYSKKYTMYHGPHREREAWPRPPLNYSHLVALALKSSPSCGLNVQQIYNFTRQHFPYFRTAPEGWKNTIRHNLCSLTCFEKVPADLEDEPDGKPRSFLWKLTEEGNRFFQEDTRVLAYARRESIKQSMRQPELIDLLFHL
Guinea_Pig	*Cavia porcellus*	ENSEMBL v.91	ENSCPOG00000001855	*foxr1*	MILHCFLYFLTLFIYFFLYKLRIVDPPVLPLEKKPNSDGDGPDIEPNLWMWVNPNIVYPPGKLEVSDPTSVADETSEVPCPQPPLEKQDHSPPRKQKRKQSFSWPAPLPFTRCTLEWRLQPTISPEGRLWSRPPLNYFHLIALALSNSPPCGLNVQQIYSFTRYVPGFPHLGLRMSTLPGQCVNRVPTGTHRCT
Pig	*Sus scrofa*	ENSEMBL v.91	ENSSSCG00000015101	*foxr1*	MGNESFLAFTTTHLPLAEQNLARYKLRIVEPPKLPLGKKPNPDNDGPDFEPNLWMWVDPNIVLPPGKLEVPQPSKGENLTSTVPSPQLSPKEEDFARYAEATVVESPSPSREQSPPRKRFASSPSTWELTEEEEAENQDDSSSVALPSPHKRAPLQSRRLRQTNSQAGRLWSRPPLNYFHLIALALRNSSPCGLNVQQIYSFTRQHFPFFRTAPEGWKNTVRHNLCFRDCFEKVPVSMQGGAPTRPRSCLWKLTEEGHRRFVEEVRALASTRLESIQQCMSQPDVMPFLFDL

Pig	*Sus scrofa*	ENSEMBL v.91	ENSSSCG00000020805	*foxr2*	MDLKLKNPDFWYSLHGQVPGLLDWDMGNEFFLPCTTDQCPLAEQNLAKYRLRVMESPKVPQEKRPSPGKDGPNSEPNLWMWVNPNIVCPFGSQEASKPSKRKNVASIFPSPQLLPKDEESNCSEAIVMESLPSSSGKQSPPQKRLTSSPSDWELTEEETGEQDNNSSVALKSPNKECFHSQKLWQGDSQERKSWPRPPLNYSHLIALALRNSPPCGLNVQEIYSFTQQHFPFFWTAPDGWKNTIRHNLCFLGSFEKVPVSLPDGANAKPRSGLWRLTEEGHRRFQEETRALASARRESIQQCMSQPDVMTSLFGL
Horse	*Equus caballus*	ENSEMBL v.91	ENSECAG00000016285	*foxr1*	MGNECFLAFTTAHLPAEKNIARYRLRIIPQLKLPLEKKPNPDKDGPDFEPNLWMWVNPNIVLPPGKLEVPEPSKGEDLTNTLPSPQSPRKEEDFANFSEATVVESLPPSSSEQSPAWKRFASSSSNWELTEEEEAEDQDDSSSVALPSPHKRAPLQSRRLRQANSQEGRLWSRPPLNYFHLIALALRNSSPCGLNVQQIYSFTRQHFPFFRTAPEGWKNTVRHNLCFRDSFEKVPVSMQGGASTRPRSCLWKLTEEGHRRFAEEARALASTRLESIQRCMSQPDVMPFLFDL
Horse	*Equus caballus*	ENSEMBL v.91	ENSECAG00000026859	*foxr2*	MDLKLKTPKFLYSLHGQVPGLMDWDMANKILLLCTTDQPVAEQDLTKYRLQVTEPRKVPQERRPSLNKDGPDVEANLWMSPNIVYSLGSQEAARPSKKTDLTSMFPSPQPLPKDKESNCSEATVMESLPSSSSESPQKQFTSSPSNWELTEEDTEEQDSNPSMTLQPPNKGECFQSQKLWQGNSQERRSWPPLNYSHLIALVSRNSPPCDLRVQEVSITQHFLFFWTAPNGWKNTISHNLCFLGSFEKATVSFQDGANARPWSGLWRLTEKHHHFQEETRALASARESIQQMRQPGVMTSLFDL
Cow	*Bos taurus*	ENSEMBL v.91	ENSBTAG00000014606	*foxr1*	VDLKLQHVDFWASLHGQVPGLLDWDMGNESFLAFATSHLPLAEQNLARYKLRIVEPPMLPLEKKPNPDKDGPDFEPNLWMWVDPNIMFPPGKLEVPEPSKGKNLTTTAPSPQPVPKEDFAKCPEATEVELLSPPGDQCSPWKRFATSLSNWELTEEKEAEDQDDRSSVALPSSHKPAPLQSRRLRQANSQEGRLWSRPPLNYFHLIALALRNSAPCGLNVQQIYSFTRQHFPFFRTAPEGWKNTVRHNLCFRDSFEKVPVSMQGGVSSRPRSCLWKLTDEGHRRFAEEARALASTRLERIQQCMSQPDVMSSLFDL
Cow	*Bos taurus*	ENSEMBL v.91	ENSBTAG00000046659	*foxr2*	MDLKLKNPEFWYSFHGQIPGLLDWDMGNEFFLPCTTDQCPLAEQNLAKYRLQVMETPKVPQERGSSPGKDRPNSEPNLWMWVNPNIACSLGSQETPEPSKKKDLAHILLSPQLLAKDEVPNCSEATVMQSLPSSSSKQSPLQKQITSFSSDRELTEDETKEKDSNSSVALQSPNKGECFQSQNLWQGNSQERKSWPRPPLNYSHLIALALRNSPPCGLNVQEIYSFTQQHFPFFWTAPYGWKNTIRHNLCFLDSFEKAPVSLQDGASARPRPGLWRLTEEGHRRFQEETRALASARRESIQQCMSHPDVMTSLFDL
Panda	*Ailuropoda melanoleuca*	ENSEMBL v.91	ENSAMEG00000000053	*foxr1*	VDLKLQHLDLWASLHGQVPGLLDWDMGNESFLAFTRCDPCSFCLLLHIIARYRLRIVEPPKVPVEKNPSPDNDGPTVEPNLWMWVNPNIVFPPGKLEVPEPRKGEDQPSTLPSPQPPPKEEDLADCSSEATAGESESLPSSSEQSPPRKRMRFASSPSTWELAEEEAADQDDSSSVALPSPHKRAPLQSQRPRQASSQEGRLWSRPPLNYFHLIALALRNSSPCGLNVQQIYNFTRQHFPFFRTAPEGWKNTVRHNLCFRDSFEKVPVSMQAGASVRPRSCLWKLTKEGHRRFAEEARALASTHLESIQQCMSQPDVMPFLFDL

Panda	*Ailuropoda melanoleuca*	ENSEMBL v.91	ENSAMEG00000018997	*foxr2*	MDIKLKNPEFWYSLHGRVPGLLDWDMGNEFFLPCTTDQCHLAEQNLAKYRLRVMEPPKVPQESRPSPDKDGPDCESNLWMWVNPNIMCPLGSQETLKPSKEKNLTRMLPYPQPLPRDEESNCSKATVMESLPTSSNKKSSPQKQCTYSPSDWEITEEKTEEQDDNCSVSLQSPNKGECFQNQKLWQGNSQERRSWPRPPLNYSHLIALALRNSPPCGLNVQEIYNFTRQHFPFFWTAPNGWKNTIRHNLCFLGSFEKAPVNLQDGTNAKPRSGLWRLTEEGHRCFQEKTRALASARRESIQQCMSQPDVMTALFGL
Opossum	*Monodelphis domestica*	ENSEMBL v.91	ENSMODG00000013351	*foxr1*	VSLLSPHKMNPKLQNREFWESLHGKVPGLLDWKMNEELKLATTTDQLPRGEMKCLNPFPISFRVKYPKPPRLSTQYVKQSYPDKNHTALEPNLWMWVDLNIVCPPPKRQTSKEPDSSQNPPSLKEEENASCSEDSVQLQPSISEQPQASTSEQELTDEEEENTSTTLPTPHTKELRQGFRRHKSTQVGVRWPRPPLNYCHLIALALKNSPPGGLNVQEIYNFTRKHFPFFQTAPEGWKNTVRHNLCFRGSFEKAPVSPDGETSRPRSYRWRLTEEGHRRFSQEARALEAAQLHSIQKCMSHPEMMSVLFDL
Bald eagle	*Haliaeetus leucocephalus*	NCBI	XP_010573382.1	*foxr1*	MYLSFQNKSFWESLHLQNGLEDWDMAEELKLTVTAEEFLQAPDQTLRHHMLKWQADPSAHGCSLPGALPDAREPGVQPHLWMWVNPSLVCPIPRTPGVDPARSNSLKALTGGATEPSSPNTSCDYSDLDCSEEDVLSSSSEGGKLTEDEDTLHVDVPVPQEMPETPELKTMPMPWKSTVLRPQSVKLKSPRQMSTEIEGGWPRPPLNYCILITLALCNSTSGSLTVQQIYQFMRQHFPFFQTAPEGWKNTIRHNLCFSSCFEKTTNFMCSEGNRKSCLWKLTPEGHRKFQEEAQALPREALDLVCQSMSEPDLMRTLFGL
Penguin	*Pygoscelis adeliae*	NCBI	XP_009321672.1	*foxr1*	MYLSFQNKSFWESLHLKNGLEDWDMAEELKLAITTEELLQALDEILRRRTLKWQADPLAHGCSLPGALPDARDKFGTWSPGVGPDGSDSPVASVAGATETSSPNTSWDYSDLDCSEEDVLSSSSEAGKLTEDEDSSRVDIPVLQEMPETPKLKAMLMPRKSAVLRPQSVKLKCPRQTSTKIEGRWPRPPLNYCILITLALCNSASGSLTVQQIYQFTRQHFPFFQTAPEGWKNTIRHNLCFSSCFEKTTGFVCGEGNRKSCLWKLTPEGRRKFQEEAQALPKEALDLVRQSMSEPELMRSLFGL
Crested ibis	*Nipponia nippon*	NCBI	XP_009467360.1	*foxr1*	MYLSFQNKSFWESLHLKNGLEDWDMAEELKLTVTTEEFLQEPGLQPHLWMWVNPSLVCPIPGSPGVEPAGSDSPVASVAGATETSSPNTSWDYSDPDCSEEDALSSSGEAGKLMEGEDASCVDIPVPQETPETPELKATLMPRKSAVLRPQSVKLKCPREMSAKIEGGWPRPPLNYCILIALALCNSASGSLTVQQIYQFTRQHFPFFQTAPEGWKNTIRHNLCFSSCFEKNTGFVCGEGNRKSCLWKLTPEGRRKFQEEAQALPKEALDLVRQSMSEPDLMRSLFGL
swan goose	*Anser cygnoides domesticus*	NCBI	XP_013042577.1	*foxr1*	MLETPKLKAMPLPQKSEIHRSQSMKLKHTRQISTKIEGGWPRPPLNYSILITLALCNSTNGSLTVQQIYQFTRQHFPFFQTAPAGWKNTIQHNLCFSSSFEKTTCFVCRNGNHKSQLWKLTPKGHQKFHEPPVPKEALDLVRIRRGPS

Chinese alligator	*Alligator sinensis*	NCBI	XP_006037673.1	*foxr1*	MATVDYKTVFLSLNKNTFWESLHLKSGLEDWDMEEELKLTNTCDQLPQAADKEVNHHMLKWQCDQQAC<br>KQSLPRERSDAPPDESNVQPHLWMWVNPNVVCPTARHLGTDATEDRSLSSSTAADISFSSPSPSLDYSNLD<br>CSEEDLLSSASEAEKFTEDEDAASVDIQLSQKMVETPKFKANLAPWKAKVSRSQSRKLKYTLQAKAKTKEIWP<br>RPPLNYCILITLALYNSAEGSLTVQQIYQFTRHHFPFFQTAPDGWKNTIRHNLCFSRSFEKTTRFVCSEGKRKA<br>RLWKLTTDGRRKFEEEMQALPTEALALVHQSMNEPELMSSLFCL
American alligator	*Alligator mississippiensis*	NCBI	XP_006266901	*foxr1*	MATVDYKTVFLSLNKSTFWESLHLKSGLEDWDMEEELKLTNTCDQLPQAADKEVNYHMLKWQCDQQACK<br>QSLPRERSDAPPDESNVQPHLWMWVNPNVVCPTARHLGTDATEDRSLSSSTAADISFSSPSPSLYYSNLDCS<br>EEDLLSSASEAGKFTEDEDAASVDIQLSQKMVETPKFKANLAPWKAKVSRSRSRKLKYTLQAKAKTKEIWPRP<br>PLNYCILITLALYNSAEGSLTVQQIYQFTRHHFPFFQTAPDGWKNTIRHNLCFSRSFEKTTRFVCSEGKRKARL<br>WKLTTDGRRKFEEEMQALPTEALALVHQSMNEPELMSSLFCL
Chinese softshell turtle	*Pelodiscus sinensis*	ENSEMBL v.91	ENSPSIG00000011857	*foxr1*	MYLNFQNKMFWESLHLKSGLEDWDMEEELKLTTTTDQFPQGSEAVRSVLMGVLHPTFSIGCCRPAARGKG<br>VKTQKSHLWMWVNPNVVCPISGCPSVDLPAPSNPEFSVADAPGFSSPSSSLNYSNLDCSEEDLLSSSSETEKF<br>TEDEAASMMDTSLFQKMKISKPKAAVTPQKPKMPRSQSKKLKYILQASTKIKGGWPRPPLNYCILITVALRNS<br>AEGSMTVQQIYQFTRQHFPFFQTAPDGWKNTIRHNLCFSSSFEKTMHFVCNEGNRKSRLWKLTPEGCRKF<br>QEEVQTLSEEALDLVSQSLSQPEQMRSLFSL
Python	*Python bivittatus*	NCBI	XP_007441556.1	*foxr1*	MLPKEDSQSSPQACRNVKVFRSRSRKLKRAQGGWPRPPLNYCILISLALSSSVDSSLTVQEIYKFTRHHFPFFR<br>TAPEGWKNTVRHNLCFSSSFEKTTDFVCLEGNRKSRLWKLTTEGRRKFQEEAQALPEDMMGLVHQSMDKP<br>ELMRSLFGL
Central bearded dragon	*Pogona vitticeps*	NCBI	XP_020649591.1	*foxr1*	MLGLPRWAVAPPSEASHAEGPGVESTMQPHLWTWVNPSLVCPIPGPVLFPPAGQEAPPAAHLLCLQEEQL<br>CSSLASQAEERGGEAPQPVLADLPQAEDAQRGQTPASRKAKTLRSRGRKLKRIQGGWARPPLNYCILISLALS<br>SSADGSLTVQEIYQFTRQHFPFFRTAPEGWKNTVRHNLCFSSSFEKTTDFVCLEGNRKSRLWKLTSEGRRKFQ<br>EEMQVLTEEVMGLVHQSMDRPELMSSLFGL
Armadillo	*Dasypus novemcinctus*	ENSEMBL v.91	ENSDNOG00000041022	*foxr1*	LPESEPETLTSRLHGQVPRLLDWDMGTSASCLLLPPLAEQKLARYRLQRVEPPKRLPEKNPRPDNDGPNIEPN<br>LWMWVNSNIMYPPEKLEATARPDKHTLLSVTPKEGDSNCSTSSWWWSHCHLSPSHLPLPQATGRQHFPF<br>FQTAPEGWKNTIHQHLCFHHSFEKVPVTMQGGSSVRPPSCLWKVTEEGHQRFEEEAGAAASTRLENIQQC<br>MSWPDMMPFLFAL

Armadillo	*Dasypus novemcinctus*	ENSEMBL v.91	ENSDNOG00000019290	*foxr2*	MDLKLKNLEFWYSLHGQVPGLLDWDMGNEFFLPHTTDQHPLAEQNLAKYQLQIMESPKLPLERRPNPDKD<br>GPDYDPNLWMWVNPNIVCSLSIQEAPNRNKEKDLTSVLPSLQPPSKDEEFTCLEATVVESLPSSSSEQYPLQK<br>RFTSFPSDWELTEEKAEEQDGNSSAALRSPDKGKCYQSQKLWQANSQERRSWPRPPLNYSHLIALALRNSP<br>PCGLNVQEIYNFTRQHFPFFWTAPDGWKNTIRHNLCFLGSFEKVPVSLQDRANARPRSCLWRLTEEGHRRF<br>QEETRALASARRESIQQCMSRPDVMTFLFDL
Xenopus	*Xenopus tropicalis*	ENSEMBL v.91	ENSXETG00000021565	*foxr1*	MLLPNPLDVALRAPKPECTFRPSLWLVVDPNLVIPCPEWINRIPPEPELTSPPLQLQRQLSNDYSTVEDSEDE<br>APTSCSDVLTDDDDSYNPWQPKHKRKKAKCLGKKLRVQKGLTQLESWPRPPLNYCNLISLALRNSEDGSLNV<br>QQIYSFVREHFPFFRIAPDGWKNTVRHNLCFSSSFEKSSGWVCADGHRRSCLWKLTRQGRRKFRNEMHALS<br>DDLLHVLRKSMKKPALMELMFGM
Xenopus	*Xenopus tropicalis*	NCBI	NP_001037918.1	*foxr1*	MYLRFANRKPYEKLHLSTALEDWDMSEELKLSITADQYFAGADDKVERYTLRRQHSTEVSPTRSEEGDYQEC<br>TFRPSLWLVVDPNLVIPCPEWINRIPPEPELTSPPLQLQRQLSNDYSTVEDSEDEAPTSCSDVLTDDDDSYNP<br>WQPKHKRKKAKCLGKKLRVQKGLTQLESWPRPPLNYCNLISLALRNSEDGSLNVQQIYSFVREHFPFFRIAPD<br>GWKNTVRHNLCFSSSFEKSSGWVCADGHRRSCLWKLTRQGRRKFRNEMHALSDDLLHVLRKSMKKPALM<br>ELMFGM
Xenopus	*Xenopus laevis*	NCBI	AAI55352.1	*foxr1*	MYLRFANRKPYEKLHLSTALEDWDMSEELKLSITADQYFAGADDKVERYTLRRQHSTELSPTRSEEGDFQEC<br>KFRPSLWLVVDPNLVIPCPEWINRIPPAPELTSPPLQLQRQLSVEYSTVEDSEDEAPTSCSDLMTDDDNDDSY<br>NPCQPKNKHKRAKCLGKKLRVQKGLTELESWARPPLNYCNLISLALRNSEDGCLNVQQIYSFVRDHFPFFRIA<br>PDGWKNTVRHNLCFSSSFEKSSGWVCADGHRRSCLWKLTRQGRRKFRNEMHALSDDLLHVLRRSMKKPAL<br>MELMFGM
Fruitfly	*Drosophila melanogaster*	ENSEMBL v.91	FBgn0015396	*jumu*	MFELEDYSSGIHEGFFSKYADAAGPSLDFYVSDSMQEMLNVDIRAEIANVVGSSSSDLTSSLDQTLEAISAINN<br>NQSNGNSSQSASYNANANFLTSSGLHASPTAKWMGSSANFWSNSDYYADLGACVNPISVMPLINSTSAG<br>MFSPKKNKTASSTQGRSGAVPSSPSAERDQHKSHLTFSPAQMKVSAGSMRRDQVMAHIPKQISVVTGTGT<br>TAPATMATNSVLQRRNSSAVDAVRKDLVTELRKAQSSPVPNSLEELGKGKGSTLLNASVGATNTIKLAPGIG<br>GLTFANSAAYQKLKQTSLVKSPGGISPGAGSNMGLKREDSNKRGLQASTTPKSIASAANSPHHQMQSNYSL<br>GSPSSLSSSSASSPLGNVSNLVNIANNNTSGAGSGLVKPLQQKVKLPPVGSPFPKPAYSYSCLIALALKNSRAG<br>SLPVSEIYSFLCQHFPYFENAPSGWKNSVRHNLSLNKCFEKIERPATNGNQRKGCRWAMNPDRINKMDEEV<br>QKWSRKDPAAIRGAMVYPQHLESLERGEMKHGSADSDVELDSQSEIEESSDLEEHEFEDTMVDAMLVEEE<br>DEEEDGDDDEQIINDFDAEDERHANGNQANNLPINHPLLGQKSNDFDIEVGDLYDAIDIEDDKESVRRIISND<br>QHIIELNPADLNATDGYNQQPALKRARVDINYAIGPAGELEQQYGQKVKVQQVIQPQQHPPTYNRRKMPL<br>VNRVI

Nematode	*Caenorhabditis elegans*	ENSEMBL v.91	WBGene00003976	*pes-1*	MTSSIKSDAPQFLLDLDNCSSLPPTPPKTASPGNSKMKGFNISDLCLDLDSSTSSSCSVSPASSFHTRSESVGQ<br>QQSGRNSPVSSSTESPTKRPKYSYNALIAMAIQSSPFKSLRVSEIYKYISSNFSYYKNQKPLQWQNSVRHNLSL<br>HKEFRKVRTLDGKGSYWAMTADLGTDVYISNNCGKLRRQKSKVAKFPPMQQHFPIPQLPTQNIHQLCMQ<br>NPQILATLLQNMYLQNMQNLQNIPMVPGFPIIPVPINPTSFHFPKSS
Sea squirt	*Ciona intestinalis*	ENSEMBL v.91	ENSCING00000018677		MLHPTQQHGVTHRTLLPMQQYPYHPYPPPPYGASSGYEGFPHQRYQQQQQPKVFPKPVYSYSCLIAMSLR<br>NSKSGCLPVADIYSFMMENFPYFKTAPDGWKNSVRHNLSLNKCFEKVGV
Lamprey	*Petromyzon marinus*	ENSEMBL v.91	ENSPMAG00000004307		CACPSVSQPYPAMRPYSNGYASHQYSPHKIHTMQGRQQKVYPKPIYSYSCLIAMALKNSKNGSLPVSDIYHF<br>MTENFPYFKTAPDGWKNSVRHNLSLNKCFEKIENKASGGSSRKGCLWTLNPAKVDKMDEEMQKWKRKDP<br>VAIRRSMANP
Coelacanth	*Latimeria chalumnae*	ENSEMBL v.91	ENSLACG00000017498		MELQVQNTVVFASLHLQTCLQDWDMDEELNLATTTDQYFQGKEDEKIRLYGRTCARRMSSPFHSPWKKM<br>YTGLHKESNVQSNLWLMVDPNLACPIGYPKVEQSTSFPVQPNPLSTATAAECPPQPSQVYPNTDDLDTDVS<br>SSISNFSDDCRKVEANPKRKNPANEDLKKNPAKPLSKKMQCLLQSSGSRKGWPRPPINYCILIALALKNSKND<br>SLNVQQIYNFTNCREHFPFFQTAPDGWKNTIRHNLCFSNSFEKTSHFVSSGRNRKSCLWRLTPEGRRKLHEEI<br>EAFPQEVLAIIQRSMNEPKMMNVMFDL
Spotted gar	*Lepisosteus oculatus*	ENSEMBL v.91	ENSLOCG00000003199		MYVQVQNRTKFLDLHLATCLKDWDMSEEIKLTTTTDQLCQDDKISSTWLCGSMWFSRCSSRQTPFGSSRST<br>LSPHLKPLFEPSLWLVVNPNLACPIKYPSPKTQPSPPGASVGIPQAPRPTRCLPPSSLSPPERAGHDDSPNSPS<br>SSSSSSSDYLLTDEEDASSPDLPAPQKPKAAKERGRRTGLAQSKRLQRILQESCNLKSGCWPRPPVNYCILIAM<br>ALSSSRAGSLNVQQIYNFTREHFPFFQTAPDGWKNTIRHNLCFSNSFEKTPQLVCSQGKRKSCLWHLTADGR<br>RRLRNEIHLLTGDSIAVLERSMTNPGMIRTLFEL
Spotted gar	*Lepisosteus oculatus*	SIGENAE	predicted in this study		MYVQVQNRTKFLDLHLATCLKDWDMSEEIKLTTTTDQLCQEPLFEPSLWLVVNPNLACPIKYPSPKTQPSPP<br>GASVGIPQAPRPTRCLPPSSLSPPERAGHDDSPNSPSSSSSSSSDYLLTDEEDASSPDLPAPQKPKAAKERGRR<br>TGLAQSKRLQRILQESCNLKSGCWPRPPVNYCILIAMALSSSRAGSLNVQQIYNFTREHFPFFQTAPDGWKN<br>TIRHNLCFSNSFEKTPQLVCSQGKRKSCLWHLTADGRRRLRNEIHLLTGDSIAVLERSMTNPGMIRTLFEL

Cod	*Gadus morhua*	ENSEMBL v.91	ENSGMOG00000009491		FFQDKKSLKMGGWPRPPVNYCILIAMALSSSHTGSLNVQQIYNFTREHFPFFQTAPDGWKNTIRHNLCFSSS<br>FCKTPYQLCREGKRKSCLWHLTLDGRHRLGDEIRTLTGESFQQLKGSMSHP
Cod	*Gadus morhua*	SIGENAE	predicted in this study		MALQFQNSSRFFELHCSTGLTDWDMDEEMKLTTTTDQFFQDEKLNDQYLAQWHYARISRRKNKYVHDQK<br>SESFVKSNLWLMVNPNLACPIKYMKRSVHVPSVVNVPVELKTPRHATERPDHSTLCTSSAAAPSCRARCAH<br>ASSSTTGFMLGDEEKAVLLDSPLYRKGPSRRKARIAKDKKSLKMGGWPRPPVNYCILIAMALSSSHTGSLNV<br>QQIYNFTREHFPFFQTAPDGWKNTIRHNLCFSSSFCKTPYQLCREGKRKSCLWHLTLDGRHRLGDEIRTLTGE<br>SFQQLKGSMSHPDVIHTLFVL
Fugu	*Takifugu rubripes*	ENSEMBL v.91	ENSTRUG00000014847		MTLQLRTKARLFDLHCSVGLTDWDMDKELKLATTTDQIYHGDDKLNEQYVVQRPAARASRRKDEFVWYDK<br>MSDTFVKPNLWLLVNPSIACPIQYGEMSSDLQTASEPAGEIQTDPAQNRVPVVQEVLLSSEYTVDPMRPNG<br>RFKSRRKGRTMAIKDFKNLKPGCWPRPPVNYCILIALALKSSHTGSLKVQQIYQFTRENFPFFQTAPDGWKN<br>TIRHNLCFNSSFRKTCNQMCRDGKRKSCFWHLTSDGQRRLLDEISTLPGETFKQLERSMSHPDVIRTLLSL
Medaka	*Oryzias latipes*	ENSEMBL v.91	ENSORLG00000019674		MTLELKTSARLLDLHCSVGLMDWDMHRELKLPTTTDQLLLDEKLKDQHAAERPSAGTLRKSDPFVWNQKT<br>PDGFKPSLWLLVNPNLVCPVQYERGAAEAVRGVAEAAERLFTDQPSSAQRETKKAEAFRRPAKSRRMGRTT<br>KVMDVSALKPGCWPRPPVNYCILIALALKSSHTGCLKVQQIYNFTRENFPFFQTAPDGWKNTIRHNLCFNSS<br>FRKTCNQLCRDGKRKSCFWHLTLDGHRRLKDEVHLLTGDTLKQLQRSMSQPDTVWSL
Medaka	*Oryzias latipes*	NCBI	NP_001153940.1	*foxr1*	MTLELKTSARLLDLHCSVGLMDWDMHRELKLPTTTDQLLLDEKLKDQHAAERPSAGTLRKSDPFVWNQKT<br>PDGFKPSLWLLVNPNLVCPVQYERGAAEAVRGVAEAAERLFTDQPSSAQRETKKAEAFRRPAKSRRMGRTT<br>KVMDVSALKPGCWPRPPVNYCILIALALKSSHTGCLKVQQIYNFTRENFPFFQTAPDGWKNTIRHNLCFNSS<br>FRKTCNQLCRDGKRKSCFWHLTLDGHRRLKDEVHLLTGDTLKQLQRSMSQPARPHESDPYVIDTLQTTSFCV<br>EVGGNENINSNHGGRCC
Platyfish	*Xiphophorus maculatus*	ENSEMBL v.91	ENSXMAG00000018797		QDLKSLKPGCWPRPPVNYCVLVALALKSSQTGSLKVQQIYNFTREHFPFFQTAPDGWKNTIRHNLCFSSSFR<br>KTCNQLCKEGKRKSCFWHLTLDGHRRLQDELHTLTGETLKMLKRSMSNP

Stickleback	*Gasterosteus aculeatus*	ENSEMBL v.91	ENSGACG00000017356		SFQDSKTLKPGCWPRPPVNYCILIALALKSSHTGGLKVQQIYNFTREHFPFFQTAPDGWKNTIRHNLCFNSSF<br>RKTCNQLCRDGKRKSCFWHLTLDGQRRLRDEIRALTGDSSRQLERSMSRP
Tetraodon	*Tetraodon nigroviridis*	ENSEMBL v.91	ENSTNIG00000018790		MTLQLRTRARLLDLHCSVGLTDWDMDKELRLATTTDQMYPDDRLSDQYVVQRPSARASRRKDEFVWYDK<br>MSDTFVKPNLWLLVNPSIACPIQYGETSSDLQTVSEPAGGTRTDPAETRNPSPTEPRPPRFQEGLPGSEYPVP<br>PTHKSPRNRFCPLSVAFQDFKNLNPGCWPRRPVNYCILIALALRSSHSGSLKVQQIYHFTRSRRTSPSYQTAPD<br>GWKNTIRHNLCFNSSFRKTCNQMCRDGKRKSCFWHLTPDGQRRLMDEISTLPRETFRQLERSMSHPVIRNV<br>IFGF
Tilapia	*Oreochromis niloticus*	ENSEMBL v.91	ENSONIG00000006931		MTLQLKTKAQLFNLHCSVGLTDWDMDKELKLASTTDQLYHGDQYVVQRPSVRTSKRKALDTEYTRMQFCH<br>RTEIICMHLHPSLIVPVHSDGQTQQMPACQNFAPPLCFLMLLFFLSCLENEHPHDTFASDFFFFFFVKNDVVS<br>CQPVKSRRKARTIKPRVRTIALTWTSFLQQAFLQDTKPLKPGCWPRPPVNYCILIALALKSSHTGSLKVQQIYN<br>FTREHFPFFQTAPDGWKNTIRHNLCFNSSFRKTCNQLCRDGKRKSCFWHLTLDGQRRLKDEIHMLTGESLK<br>QLEKSMSHPVSDIIRSLFPM
Bowfin	*Amia calva*	SIGENAE	predicted in this study		MFLQLQNRDSFLDLHQTTCLNDWDMNEEIKLATTTDQFYQDEKRNDQYLVQWHYARFSRRSSSGKDDSPS<br>QSPSEMDEPQIQPSLWLVVNPCLVCPIKYPCQMTQVSPPAVTTWSPLTPSPALSPLPPLEEPSLDESLLSGSS<br>SSEFMFTDDDDTSSVDVPVPRKPKTTRKPKAPKERGRKPGPAQSRRLARVLQESGNLNKGGWPRPPVNYCI<br>LIAMALNSSRCGSLNVQQIYNFMREHFPFFQTAPDGWKNTIRHNLCFSNSFEKTPQLVSSQGKRKSCLWHL<br>TLDGRRRLRNEIHTLTGDSLRVLRRSMAEPDMISTLFEL
European eel	*Anguilla anguilla*	SIGENAE	predicted in this study		MFLHLQNRSNFLDLHLKTCLNDWDMNEEIKLTTTTDQFFQDEKRNDQYLVQWHYARIARRTSSGKDDLSCL<br>PSQESEEPQVQPNLWLMVNPTLACPIKYPKGSPTLTETSTIPEQHPPVMPVPAPTLAPVPSAHPPSPVVERA<br>LDESIHDASSSSEYLLTDEDDASSVDVPICRKVKSGGRKGKSPKDRVRKLNLAQNKRLQRVLQESNNLKSGA<br>WPRPPVNYCILIAMALNSSRTGSLNVQQIYNFTREHFPFFQTAPDGWKNTIRHNLCFSNSFRKTPQQVSSEG<br>KRKSCLWHLTVDGRRRLRNEIHTLTGESFTVLRMSMNNPGTVL
Butterflyfish	*Pantodon buchholzi*	SIGENAE	predicted in this study		MTRSWQRDAPRGFHKGHARLRHLWLCLELGGAARTGDGKMFLQVQNRSTFLDLHLTTALSDWDMSEEV<br>KLATTTDQYCHDEKCGDQYLAQWHYARNSRRASGTEEGAGQQSEPQVQPNLWLMVNPNLACPIKYPDN<br>VLGARARVSPSPKLSPIGSAPPGNVVCTRPLSPTDRLTLDSPLQDASSSSEYMLTDEDDASSVDVPICRKVKSC<br>WRARVPKERVRKLGLAQSKRLQRVLQESNNLKNGGWPRPPVNYCILIAMALSSSHSGSLNVQQIYNFTREH<br>FPFFQTAPDGWKNTIRHNLCFSNSFKKTPQQVPSEGKRKSCLWHLTGDGRQRLRDEIQTLTGESFRVLKRSM<br>HDPDMIQTLFDL

Sweetfish	*Plecoglossus altivelis*	SIGENAE	predicted in this study		MSLQFQARRRFLELHLSTGLNDWDMSEEINLTTTTDQFYQDNKWNDQYIVQWHYARIARRKEDLLSQNTH<br>KSETQVHPNLWLMVNPNLACSIEYPKALKAPSRPKMQAQQTRPNPQSMGTPIQPRQIPQEISPIDIACLEDS<br>HHEVASSSEYMMTDEDDASSVDVPIYRKAKSTRKGKVPTKAGMRKLILAQNKRLQRVLQDSNNLKSGAWP<br>RPPVNYCILIAMALSSSHAGSLNVQQIYNFTREHFPFFQTAPEGWKNTIRHNLCFSSSFRKTPQQVCSEGKRK<br>SCLWHLTLDGHRRLRHEIHTLNVDSFRVLKRSMNDPDMIRTLFEL
Allis shad	*Alosa alosa*	SIGENAE	predicted in this study		MFLRFGNIDRFLDLHLTTGLTDWDINEEINLTTTTDQYHQDGQRNEYLIQWHYARMSRRTSTAEIPCQYSKQ<br>SDENDIEPNLWLIVNPNLVCPMHPSRLTPTKAYVGTPNIDHQFINTHLPYEPLTPVSPEVLDKSPSDTFFTSKR<br>MVSDDESSAGLEICRKMKVTRQSRVPKEGERACRTGQTRGRGRGRPPRRVHKDSDSFAFRSWAWPRPPV<br>NYCLLIAMALGSCRTGSLNVQQIYSFTREHFPFFQTAPDGWKNTIRHNLCFSTSFKKTPQQVSSGGERKSCL<br>WHLTSDGRMRHRSEIQMLSDDAIKQLTRSMKYPDLIQELLDL
Arowana	*Osteoglossum bicirrhosum*	SIGENAE	predicted in this study		MFLHIENRSSFLELHLKMRLADWDMSEEVKFATTTDQFCQDGTQSEQGAARWDGAKLSKRDDDDDKEEE<br>EARGEPHEGSGSQVEPSLWLMVNPNVACPIKYPKQAARDPTPAWPMHKGPPGVSSAVFSQSLIPHVRPPS<br>PAEEAFPDASLHEASSSSEYLATDEDSASPLDVQVCCEAKWSRGLKLPKEKIRKLGMAQSKRLQRVLQESNYL<br>KNGEWPRPPVNYCILIAMALNSSHCGSLNVQQIYNFTREHFPFFQTAPDGWKNTIRHNLCFSNSFKKTPQQ<br>VSSEGKRKSCLWHLTLDGRRRLRDEIYTLSGESFRVLKRSMNNPDLIQTLFDL
Zebrafish	*Danio rerio*	ENSEMBL v.91	ENSDARG00000004864	*foxr1*	MFLQLQSKSKFLELHLTSGLHDWDMNEEIKLTTTTDQFYHDEKRTDQYLAQWHYARISRRSSLPAAHRSLEL<br>PHRDPEIQPNLWLMVNPSLACPIKYPSNLPKMTTPPKPAVPRSSTPVSDPPLLPDETCLNESVQDTSVSSEYQ<br>LTDEDDASSVDVPICRKVKGTRKGRTPKANTRRLGLTQSRRLQRALQDSMSLKSGVWPRPPVNYCILIAMAL<br>SSSRSGSLNVQQIYNFTREHFPFFLTAPDGWKNTIRHNLCFSNSFKKTPQQVSGDGKRKSCLWHLTLDGRQR<br>LRDEIHTLTEDSFRLLRRSMNYPDMIPALLEL
Zebrafish	*Danio rerio*	NCBI	NP_001096594.1	*foxr1*	MFLQLQSKSKFLELHLTSGLHDWDMNEEIKLTTTTDQFYHDEKRTDQYLAQWHYARISRRSSLPAAHRSLEL<br>PHRDPEIQPNLWLMVNPSLACPIKYPSNLPKMTTPPKPAVPRSSTPVSDPPLLPDETCLNESVQDTSVSSEYQ<br>LTDEDDASSVDVPICRKVKGTRKGRTPKANTRRLGLTQSRRLQRALQDSMSLKSGVWPRPPVNYCILIAMAL<br>SSSRSGSLNVQQIYNFTREHFPFFLTAPDGWKNTIRHNLCFSNSFKKTPQQVSGDGKRKSCLWHLTLDGRQR<br>LRDEIHTLTEDSFRLLRRSMNYPDMIPALLEL

Zebrafish	*Danio rerio*	ENSEMBL v.91	ENSDARG00000011879	*foxN1*	MSSEPQGLSFLSISSSSSSPSPSAGQLQSFDSLESPCFQMPESQCHQSNGGEGFVPYSQRKSDPYSQKSAERF<br>RRHSVDGSPVGRDCDLTENSHYHPYRRQCSEGAISEGQPFSCVRRAGLAREIASADGLEEQSSWTTLGTDVE<br>TTSIMGTRRPYSEPQESSEEPPGYTTVNHQAYSAISPLQHQYYPSRGIDTVSHYYNQSLSTQTSQDSSPQPLY<br>PKPVYSYSILIFLALRNSKTGSLPVSEIYSFMTEHFPYFKTAPDGWKNSVRHNLSLNKCFEKVENKNGNSSRKG<br>CLWALNPAKVEKMQEELHKWRRKDPLTVRRSMARPEELERLLGERPEKLKSHFSLPSSHTHSQPFRVNMHP<br>AYGHQPVHENRILNQKALYNPLTSQNAVLPPPYISPDPLAFQYYSPATHQPSVGHPSSPRTGSLDSPLPAHTP<br>PSYSTALQAGHSGTASMQELLLDGEINNDVDALNPSLTDLQLHGSLWEELRNDSLAPDSLIVMDSASSLAQL<br>SPPPARDSAGVCAETEDGVQGSISELYLSSFYTTAFTSAENMPGLISSSANTAIPLL
Zebrafish	*Danio rerio*	ENSEMBL v.91	ENSDARG00000012833	*foxN3*	MGPVMPPSKKPESSGVSVASGLSQFYQASSITRALQEAEEMDLALPTIKLEKGSVEDEELTNLNWLHESKNLL<br>NSFGDPVLRSVSPVQDLDDDTPPSPAHSDLPYDAKQNPNCKPPYSFSCLIFMAIEDAPSKRLPVKDIYNWILE<br>HFPYFANAPTGWKNSVRHNLSLNKCFKKVDKDRSQSIGKGSLWCIDPEYRQNLIQALKKTPYHPYSQVFTTP<br>PTSPQAYQSMSSPPIWPGSPFYRKNKGVLLQVPQGVIQNGARMMSQGLFPGIRPLPINPIVSRTAAVRSVL<br>GYRITSDTEPTQNSPLATSDPKEDHTYSFAKSSGSDAEEEAQCDGSELNFHTNETGSDAEDDDKSKIKKEPKE<br>WSVDALALAQHKKRQLLTKVKQRVASDTLPLKKRRTEKPPESDDEEMKEAAGSLLHLAGVRACLNNITNRTA<br>KGQKEQK
Cave fish	*Astyanax mexicanus*	ENSEMBL v.91	ENSAMXG00000007405	*Predicted foxr1*	LQARERFLSLHLSTGLHEWDMSEEIKLSTTTDQFHRDDKRNEQYLAQWHYARISRRTLAGADSTHLAQPEDC<br>TESIIQPSLWLIVNPNLACPIKYPTVKKSSLLKLTVDTPVPSPSPPVPVQNLHILPLPDQTCLNESLHDTSLSSEY<br>QLTDDDTSSVDVPICRKVKGARKGRVPKEPSSRRLGLAQSRRLQRVLQDSCNLKNGAWPRPPVNYCILIAMA<br>LSSSRSGSLNVQQIYNFTRDHFPFFITAPDGWKNTIRHNLCFSNSFKKTPQQVTGEGKRKSCLWYLTLDGRRR<br>LRNEIHTLTVDSFRMLKRSMNYPDMIQALFEL
Panga	*Pangasius hypophthalmus*	SIGENAE	predicted in this study		MFLKLRASEKFLDLHLTTGLHDWDMNEEINLTTTTDQFSQDEMRNEQYLAQWHYARISRRTLPEELHQLSP<br>ANCEESVIQPNLWLMVNPCLACPIKYPVRDKKPSLPKLTIDRSASGPALAVPGQNLHLLPLSDETGLDESLHD<br>ASLSSEYQLTDDDASSEDVAIRHKVKGTRRGRAPKEPSSHRLGLAQSRRLQRVLQDSRNLKSGAWPRPPVNY<br>CILIAMALSSSRSGSLNVQQIYNFTREHFPFFLTAPEGWKNTIRHNLCFSNSFRKTPQQVTGDGKRKSCLWHL<br>TLDGRRRLRNEIHTLTADSFRMLKRSMNYPDMIQALFEL
Northern pike	*Esox lucius*	NCBI	XP_019904158.1	*foxr1*	MSLHFQTRSRFLELHLSTRLYDWDMEEEIKLTTTTDQFFQDDKRNDQYLVQWHYARISRRKEDLSYQNREKT<br>ESPVEPNLWLMVNPNLACPIKYPKKVTKILSPKTPLEHPAPVQPTVNHVKRMSAQNVFPASHIEGTCLEESL<br>HDASSSSEYMLTDEDDTSSVDLPICRKLKTARKAKASKREPRKLSLAHSKRLQQVLQDNNNMKNGAWPRPP<br>VNYCILIAMALSSSRTGSLNVQQIYNFTREHFPFFQTAPDGWKNTIRHNLCFSNSFRKTPQQVCSEGKRKSCL<br>WHLTPDGRRRLRDEIHTLTGDSYKVLKRSMNNPEMIQTLFEL

Northern pike	*Esox lucius*	SIGENAE	predicted in this study		MSLHFQTRSRFLELHLSTRLYDWDMEEEIKLTTTTDQFFQDDKRNDQYLVQWHYARISRRKEDLSYQNREKT<br>ESPVEPNLWLMVNPNLACPIKYPKKVTKILSPKTPLEHPAPVQPTVNHVKRMSAQNVFPASHIEGTCLEESL<br>HDASSSSEYMLTDEDDTSSVDLPICRKLKTARKAKASKREPRKLSLAHSKRLQQVLQDNNNMKNGAWPRPP<br>VNYCILIAMALSSSRTGSLNVQQIYNFTREHFPFFQTAPDGWKNTIRHNLCFSNSFRKTAQQVCSEGKRKSCL<br>WHLTPDGRRRLRDEIHTLTGDSYKVLKRSMNNPEMIQTLFEL
Eastern mudminnow	*Umbra pygmae*	SIGENAE	predicted in this study		MSLHFRTRSRFLELHLSTRLYDWDMDEEIKLTTTTDQFYQDDKRNDQYLVQWHYARISRRKDDLSCQNRVK<br>TESPIQPNLWLMVNPNLACPIKYPKTVTKILSPKIRLEHPEPVQPPDNHGKINSIANVLPALPFDAESLPGAISS<br>SEYMQTDEETSSVDIPICRKVKTTRKAKVPKYGPRKLSLAQSKRLQRILQDKNILKSGAWPRPPVNYCILIAMA<br>LSSCRSGSLNVQQIYKFTREHFPFFQTAPDGWKNTIRHNLCFSNSFRKTPQQVSSQGKRKSCLWHLTLDGRQ<br>RLRDEIHTLTGDSYRVLKRSMNNPDMIRALFDL
American whitefish	*Coregonus clupeaformis*	SIGENAE	predicted in this study		MCLQFQTRSRFLELHLSTSLNDWDMDEEIKLTTTTDQFYQGDKRNDQYLVQWHYAKISRRKDDLSCQNYQ<br>KSESPMQPNLWLMVNPNLACPIKYPKTVTKILSPKMPLKHLAPVPKPTVTHVKPKSIPNVLPVSPTADTCLAD<br>SLHDASSSEYMLTDEDDSSSVDVSVCRKAKTVHKAKALKGGPRKLSLAQSKRLQRVLQDSNNLKSGAWLRPP<br>VNYCILIAMAIGSNRTGSLNVQQIYNFTREHFPFFQTAPDGWKNPIRHNLCFSNSFRKTPQQVCSEGKRKSCL<br>WHLTLEGRRRLRDEIHTLTGDSYRVLKRSMNNPDMIQTLFEL
Brook trout	*Salvelinus fontinalis*	SIGENAE	predicted in this study	*foxr1*	MSLQFQTRSRFLELHLSNSLNDWDMDEEIKLTTTTDQFYQAEDKRNDQYLVQWQYARISRRKDDLSFQNY<br>QKSDSPIQPNLWLMVNPNLACPIKYPKKVTKAPVPKLTVTHVKPKSIPNVLPVSPTEDTCLADSLHDASSSEY<br>MLTDEDDASSLDVPICRKVKTARKAKAPKGGPRKLSLAQSKRLQRVLQDSNNLKSGAWLRPPVNYCILIAMA<br>IGSSRTGSLNVQQIYNFTREHFPFFQTAPDGWKNTIRHNLCFSNSFRKTPQQVCSEGKRKSCLWHLTLEGRR<br>RLRDEIHTLTGDSYKVLKRSMNNPDMIQTLFAL
Brook trout	*Salvelinus fontinalis*	SIGENAE	predicted in this study		MSLQFQTRSRFLELHLSNSLNDWDMDEEIKLTTTTDQFYQEDKRNDQYLVQWQYARISRRKDDLSFQNYQ<br>KSDSPIQPNLWLMVNPNLACPIKYPKKVTKAPVPKLTVTHVKPKSIPNVLPVSPTEDTCLADSLHDASSSEYM<br>LTDEDDASSLDVPICRKVKTARKAKAPKGGPRKLSLAQSKRLQRVLQDSNNLKSGAWLRPPVNYCILIAMAIG<br>SSRTGSLNVQQIYNFTSNSFRKTPQQVCSEGKRKSCLWHLTLEGRRRLRDEIHTLTGDSYKVLKRSMNNPDM<br>IQTLFAL
Brown trout	*Salmo trutta*	SIGENAE	predicted in this study		MRMSLQFQTRSRFLELHLSNSLNDWDMDEEIKLTTTTDQFYQGDKQNDQYLVQWQYARISRRKDDLSFQ<br>NYQKSSDSPIQPNLWLMVNPNLACPIKYPKKVTKILSPKVPLKHLAPVPKPTVPHVKPKSIPNVLPVSPTEDTC<br>LADSLHDVSCSEYMLTDEDDASSVDVPVCRKVKTARNAKAPKGGPRKLSLAQSKRLQRVLQDSNNLKSGAW<br>LRPPVNYCVLIAMAIGSSRTGSFNVQQIYNFTREHFPFFQTAPDGWKNTIRHNLCFSNSFRKTPQQVCSEGK<br>RKSCLWHLTLEGRRRLRDEIHTLTGDSYKVLKRSMNNPDMIQTLFAL

Rainbow trout	*Oncorhynchus mykiss*	NCBI	XP_021479605.1	*foxr1-like*	MSLQFQTRSRFLELHLSNSLNDWDMDEEIKLTTTTDQFYQAGDKRNDQYLVQWQCARISRRKDDLSFQNYQKSVEDSPIQPNLWLMVNPNLACPIKYPKKVTKILSPKVPLKHMAPVPKPTVTHVKPKSIPNVLPVSPPEDTCLADSRHDVSSSEYMLTDEDDASSVDVPVCRKVKTARKAKAPKGGPRKLSLAQSKRLQRVLQDSNNLKSGAWLRPPVNYCILIAMAIGSSRTGSLNVQQIYNFTREHFPFFQTAPDGWKNTIRHNLCFSNSFRKTPQQVCSEGKRKSCLWHLTLEGRRRLRDEIHTLTGDSYKVLKRSMNNPDMIQTLFAL
Rainbow trout	*Oncorhynchus mykiss*	SIGENAE	predicted in this study		MSLQFQTRSRFLELHLSNSLNDWDMDEEIKLTTTTDQFYQGDKRNDQYLVQWQCARISRRKDDLSFQNYQKSVEDSPIQPNLWLMVNPNLACPIKYPKKVTKILSPKVPLKHLVPVPKPTVTHVKPKSIPNVLPVSPPEDTCLADSRHDASSSEYMLTDEDDASSVDVPVCRKVKTARKAKAPKGGPRKLSLAQSKRLQRVLQDSNNLKSGAWLRPPVNYCILIAMAIGSSRTGSLNVQQIYNFTREHFPFFQTAPDGWKNTIRHNLCFSNSFRKTPQQVCSEGKRKSCLWHLTLEGRRRLRDEIHTLTGDSYKVLKRSMNNPGEFQGGTVVELHTEPLN
European whitefish	*Coregonus lavaretus*	SIGENAE	predicted in this study		MCLQFQTRSRFLELHLSTSLNDWDMDEEIKLTTTTDQFYQGDKRNDQYLVQWHYAKISRRKDDLSCQNYQKSESPMQPNLWLMVNPNLACPIKYPKTVTKILSPKMPLKHLAPVPTPTVTHVKPKSIPNVLPVSPTADTCLADSLHDASSSEYMLTDEDDSSSVDVSVCRKAKTVHKAKALKGGPRKLSLAQSKRLQRVLQDSNNLKSGAWLRPPVNYCILIAMAIGSNRTGSLNVQQIYNFTREHFPFFQTAPDGWKNTIRHNLCFSNSFRKTPQQVCSEGKRKSCLWHLTLEGRRRLRDEIHTLTGDSYRVLKRSMNNPDMIQTLFEL
Coho salmon	*Oncorhynchus kisutch*	NCBI	XP_020340186.1	*foxr1-like*	MSLQFQTRSRFLELHLSNSLNDWDMDEEIKLTTTTDQFYQAGDKRNDQYLVQWQYARISRRKDDLPFQNYQKSVEDSPIQPNLWLMVNPNLACPIKYPKKVTKLLSPKVPLKHLAPVPKPTVTHVKPKSIPNVLPVSPTEDTCLADSPHDASSSEYMLTDEDDTSSVDVPVCRKVKTARKAKAPKGGPRKLSLAQSKRLQRVLQDSNNLKSGAWLRPPVNYCILIAMAIGSSRTGSLNVQQIYNFTREHFPFFQTAPDGWKNTIRHNLCFSNSFRKTPQQVCSEGKRKSCLWHLTLEGRRRLRDEIHTLTGDSYKVLKRSMNNPDMIQTLFAL
Grayling	*Thymallus thymallus*	SIGENAE	predicted in this study		MSLQFQTRSRFLELHLSTSLNDWDMDEEIKLTTTTDQFYQGAKRNDQYLVQWHYARISRRKDDISCQNYQKSESPMQPNLWLMVNPNLACPIKYPKKVTKILSPKMPLKHLAPVPKSIPNVLPVSPTEDTCLANSVHDASSSEYMLTDEDDASSVDVPVCRKVKTARKAKAAKGGPRKLSLAQSKRLQRVLQDSNNLKSGAWLRPPVNYCILIAMAISSSRTGSLNVQQIYNFTREHFPFFQTAPDGWKNTIRHNLCFSNSFRKTPQQVCSEGKRKSCLWHLTLEGRRRLRDEIHPLTGDSYRVLKRSMNNPDLIQTLFAL
Atlantic salmon	*Salmo salar*	NCBI	XP_013992597.1	*Predicted foxr1-like*	MRMSLQFQTRSRFLELHLSNSLNDWDMDEEIKLTTTTDQFYQAGDKQNDQYLVQWQYARISRRKDDLSFQNYQKSDSPIQPNLWLMVNPNLACPIKYPKKVTKILSPKVPLKHLAPVPKSTVPHVKPKSIPNVLPVSPTEDICLADSLHDASSSECMLTDEDDASSVDVHVCRKVKTARNAKAPKGGPRKLSLAQSKRLQRVLQDSNNLKSGAWLRPPVNYCVLIAMAIGSSRTGSLNVQQIYNFTREHFPFFQTAPDGWKNTIRHNLCFSNSFRKTPQQVCSEGKRKSCLWHLTLEGRRRLRDEIHTLTGDSYKVLKRSMNNPDMIQTLFAL

European perch	*Perca fluviatilis*	SIGENAE	predicted in this study		MTLQLKTKARLLDLHCSVGLTDWDMDKELRLATTTDQFYHDDKLNDQYVVQRPSARASRRKDEFIWYDKTSDTFVKPNLWLLVNPSIACPIQYGENTADLQTLCEPAEEIQTPLLNPAETQRQLATELHMFDPHGAPLQEELLHSTEYMNEDVSCRPAKNRRKGRNTKAKDNKILKPGCWPRPPVNYCILIALALKSSHTGSLKVQQIYNFTREHFPFFQTAPDGWKNTIRHNLCFNNSFRKTCNQVFRDGKRKSCFWHLTLDGHRRLKDEIRTLTGESLKQLERSMSRPDVIQSLFAL
