# Supplementary material for: foxr1 is a novel maternal-effect gene in fish that is required for early embryonic success
Source: PeerJ. 2018 Aug 23;6:e5534. doi: 10.7717/peerj.5534 (PMC6109588; doi:10.7717/peerj.5534)
Supplement: Supplemental Information 2 — For each gene, primer sequence is provided. [file peerj-06-5534-s002.pdf]

Supplemental Data 2: Primers used in this study.

**Gene**	**Accession number**	**Product size (bp) [WT or mutant]**	**Primer sequence (5' – 3')**
foxr1 genotyping forward	ENSDARG00000004864	400 or 160	ATCCGCCATAACCTGTGCTT
foxr1 genotyping reverse			GCAGGTTCATTTCTCCAGCC
foxr1 qPCR forward		57 or 0	AGAAAGTCCTGTCTGTGGCA
foxr1 qPCR reverse			CTCGTCTCTCAGTCTCTGCC
cloning foxr1 forward		729	TTACCACGATGAGAAGCGGA
cloning foxr1 reverse			TGCCACAGACAGGACTTTCT
p21 forward	ENSDARG00000076554	198	GCCGCTGGAAAGGAAAACAT
p21 reverse			TGTGTGTGTGTGTGTCTCCT
p27 forward	ENSDARG00000054271	139	GGGAATCACGACTGTAGGGT
p27 reverse			TTCGGGTCACTTCATCCACA
rictor forward	ENSDARG00000002020	197	CCAGCTCTAGACACAACAGC
rictor reverse			GGAGAGGGAGATCAGGAAGC
gfp		152	AGACGTTGTGGCTGTTGTAG
vasa	AF461759.1		TTAGACATGACCGGAAGAGTTG
18S rRNA forward	KY486501.1	118	CGGAGGTTCGAAGACGATCA
18S rRNA reverse			AAGACTCGTGGTTTCCCGCA
β-actin forward	NM_181601.4	200	CCGTGACATCAAGGAGAAGCT
β-actin reverse			TCGTGGATACCGCAAGATTCC
EF1 α forward	NM_199949.2	160	CTTCCTCCAAGCCCAGGACT
EF1 α reverse			TAACGTCATCAGCCTGAGAGG
