# Supplementary material for: foxr1 is a novel maternal-effect gene in fish that is required for early embryonic success
Source: PeerJ. 2018 Aug 23;6:e5534. doi: 10.7717/peerj.5534 (PMC6109588; doi:10.7717/peerj.5534)
Supplement: Supplemental Information 3 — Sequence positivity and similiarity is shown for the proteins of the different species. [file peerj-06-5534-s003.pdf]

	Zebrafish FoxR1		FoxR1 : FoxR2	
	Positivity	Similarity	Positivity	Similarity
Human FoxR1	34%	50%	56%	72%
Human FoxR2	32%	48%		
Mouse FoxR1	37%	53%	47%	62%
Mouse FoxR2	33%	49%		
Rat FoxR1	34%	49%	45%	59%
Rat FoxR2	32%	48%		
Pig FoxR1	34%	50%	66%	77%
Pig FoxR2	35%	48%		
Horse FoxR1	39%	53%	55%	66%
Horse FoxR2	29%	41%		
Cow FoxR1	35%	50%	60%	72%
Cow FoxR2	33%	48%		
Panda FoxR1	33%	46%	60%	73%
Panda FoxR2	34%	48%		
