# Supplementary figures and images for: foxr1 is a novel maternal-effect gene in fish that is required for early embryonic success
Source: PeerJ. 2018 Aug 23;6:e5534. doi: 10.7717/peerj.5534 (PMC6109588; doi:10.7717/peerj.5534)

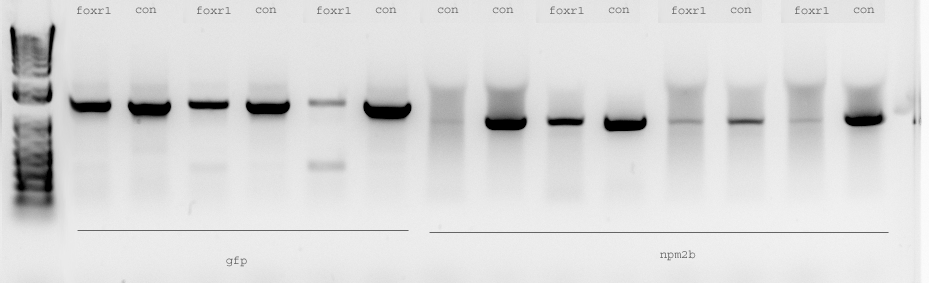

Supplement: Supplemental Information 5 [file peerj-06-5534-s005.tif]
